# Nanocompartments work as tiny loudspeakers to amplify cell signalling behaviour

**DOI:** 10.1038/s42003-020-01352-y

**Published:** 2020-10-23

**Authors:** Anam Akhtar

**Affiliations:** Communications Biology, https://www.nature.com/commsbio

## Abstract

Aberrant cell signalling has been associated with a number of diseases. Belluati and coworkers make use of dual polymer nanocompartments encapsulating different enzymes, that function in unison as in a native signalling cascade. Their functionality is integrated into native cell metabolism and physiology, using substrates already present in the extracellular medium. They succeed in amplifying a natural signalling cascade and influencing cellular homoeostasis.

The most complex molecular machines are found within cells, which need constant communication in order to function smoothly. Malfunctioning of a communication thread results in disorders, so approaches to control the cell’s own signalling pathways are of considerable interest to treat various diseases^1^.

The cascade reaction mediated by nitric oxide synthase (NOS) and guanylyl cyclase (GC) is one such pathway where malfunction of one or both enzymes has a detrimental effect on homoeostasis of organisms and affects the cell’s growth and differentiation capacity. NOS produces nitric oxide, which binds to the heme moiety of its signalling partner, GC. This induces the transition to active sGC, which converts gaunosine-5′-triphosphate (GTP) to cyclic 3,5-guanosine monophosphate (cGMP). cGMP acts as a second messenger in a variety of processes, through intra- and inter signalling cascades. Aberrant cGMP signalling results in a number of diseases, including muscular and retinal dystrophies.

A recent study led by Professor Cornelia Palivan at the University of Basel reports loading of these enzymes into nanocompartments which can enter cells and get integrated into the native signalling process. For encapsulation, they use polymer nanoparticles, which protect enzymes from immature degradation and allow for more precise kinetic regulation. To permeabilise the membranes of these catalytic nanocompartments (CNC) for the substrate/product traffic, they insert bacterial Outer membrane protein F (OmpF) into their shells. By encapsulating the two enzymes NOS and GC separately into such CNCs and functionally coupling them (where the product of an enzymatic reaction happening in one CNC starts the reaction in the other), they were able to amplify the cGMP pathway in HeLa and smooth muscle cells. As NOS and GC are already present in cells albeit in low quantities, this arrangement “amps” up the needed signal. The effect of cGMP production is sensed by measuring cytoplasmic calcium levels over time, which shows that the different CNCs, working in tandem, elicit the highest response.

This study brings the potential of catalytic nanoparticles closer to reality where they can be directly integrated within cellular physiology, boost metabolic processes while using the substrate already present in the extracellular medium.
